# Drug Management of Visceral Pain: Concepts from Basic Research

**DOI:** 10.1155/2012/265605

**Published:** 2012-04-24

**Authors:** Mellar P. Davis

**Affiliations:** ^1^Cleveland Clinic Lerner School of Medicine, Case Western Reserve University, Cleveland, OH 44195, USA; ^2^Solid Tumor Division, Harry R. Horvitz Center for Palliative Medicine, Taussig Cancer Institute, USA

## Abstract

Visceral pain is experienced by 40% of the population, and 28% of cancer patients suffer from pain arising from intra- abdominal metastasis or from treatment. Neuroanatomy of visceral nociception and neurotransmitters, receptors, and ion channels that modulate visceral pain are qualitatively or quantitatively different from those that modulate somatic and neuropathic pain. Visceral pain should be recognized as distinct pain phenotype. TRPV1, Na 1.8, and ASIC3 ion channels and peripheral kappa opioid receptors are important mediators of visceral pain. Mu agonists, gabapentinoids, and GABAB agonists reduce pain by binding to central receptors and channels. Combinations of analgesics and adjuvants in animal models have supra-additive antinociception and should be considered in clinical trials. This paper will discuss the neuroanatomy, receptors, ion channels, and neurotransmitters important to visceral pain and provide a basic science rationale for analgesic trials and management.

## 1. Introduction

Normal individuals do not perceive signals emanating from their intestinal tract; however, enteric and extrinsic visceral afferents become hypersensitive in pain-processing disorders such as functional bowel syndromes or in diseases associated with inflammation such as inflammatory bowel disease and pancreatitis. Both inflammatory bowel disease and cancer-related metastases to viscera may produce persistent pain despite resolution of the underlying disease state [1]. Unexplained abdominal pain accounts for 40% of gastroenterology practice in the United Kingdom. Most abdominal pain is due to functional gastrointestinal disorders; irritable bowel syndrome, and functional dyspepsia [2]. Ten to 40% of the normal population will have complaints of abdominal cramps or pain. Most use over-the-counter medications usually antispasmodics and or antacids [3]. Inflammatory bowel disease causes visceral hypersensitivity, intestinal stenosis, anorectal urgency, fistula, and abscess. One-third to half of individuals with inflammatory bowel will have disabling visceral symptoms such as pain or colic or symptoms that resemble the irritable bowel syndrome [4, 5]. Individuals with Crohn's disease in remission frequently have irritable bowel syndrome symptoms as a result of persistent visceral hypersensitivity which may mislead both patients and clinicians to believe that the Crohn's disease is active [6–9]. Hence, distinguishing functional from organic visceral pain can be a challenge. The duration of pain is longer with functional bowel disorders, whereas organic etiologies produce nocturnal pain and are frequently associated with weight loss and constitutional symptoms [3].

Visceral pain accounts for 28% of cancer-related pain. It is often accompanied by other pains such as neuropathic or somatic pain. Visceral pain in cancer patients may also be the result of treatment complications or comorbid diseases [10]. Causes of cancer-related visceral pain include hepatic metastases with extension to the hepatic capsule, biliary obstruction, pancreatitis as well as pancreatic primaries and peripancreatic nodal enlargement, retroperitoneal adenopathy from metastases, and visceral organ obstruction such as small bowel or colon obstruction, mesenteric infiltration, and peritoneal implants of cancer. Complications include not only intestinal obstruction by perforation and intussusceptions, but also visceral pain is described as pressure-like, intermittently squeezing or cramp, not well localized, vague in character, and difficult for patients to describe. Visceral pain is frequently accompanied by nausea, vomiting, and sweating. Pain, particularly if severe, is often referred to distant somatic (superficial) sites [11]. Treatment recommendations for visceral pain have been the same for somatic pain, yet visceral pain processing is distinctly different from somatic nociception and as a result should perhaps be treated differently from somatic pain (Table 1).

## 2. Neuroanatomy

### 2.1. Vagus Innervation

This sensory system of the gastrointestinal tract consists of intrinsic (enteric) sensory afferents and extrinsic (vagus, spinal, and pelvic) afferents. The intrinsic system functions independently of the CNS. Neurons are directly exposed mechanical forces and the chemical environment which is unlike somatic afferents neurons. Enterochromaffin cells within the mucosa and enteroendocrine cells release serotonin, cholecystokinin, orexin, and leptin which modulates and regulates motor activity [12]. The submucosal enteric plexus and myenteric plexus have a high degree of synaptic interactions which can be either inhibitory or stimulatory for the purpose of regulating gastrointestinal motility and peristalsis. Both plexuses received input from parasympathetic and sympathetic efferents. Vagal afferents are largely made up of neurons which interact with the submucosal and myenteric plexus and allow “crosstalk” between intrinsic and extrinsic systems [12]. The intrinsic system contains ganglia and interstitial cells of Cajal which act as pacemakers and gauges for muscle tension. Certain ion channels such as the transient receptor potential (TRP) group of ion channels which have a major role in pain processing are not found on intrinsic enteric neurons [12].

 The vagus receives input largely from the mucosa and muscle enteric afferents as do the pelvic extrinsic afferents. Cell bodies of the vagus are found in the jugular and nodose ganglia; both ganglia are morphologically, biochemically, and functionally different from one another. Expression of ion channels and signal transduction are distinctly different between the two ganglia [12]. Vagal sensory afferents outnumber efferents 10 to 1 in both ganglia. Both project to the nucleus tractus solitarius [13]. From the nucleus tractus solitarius, second-order afferents project to the parabrachial nucleus and thalamus. Vagal neurons process physiologic information such as the nature and content of luminal constituents and presence or absence of motor activity and in response generate nonpain but potentially noxious symptoms such as nausea, vomiting, and early satiety [13]. Vagal afferent terminals are found in close proximity to enterochromaffin cells and are responsive to regional serotonin by way of certain serotonin (5HT1 5HT 3, 5HT4, and 5HT7) receptors. Ghrelin, released from gastric endocrine cells, binds to receptors on the vagus and nodose ganglia. Ghrelin blocks mucosal and muscle enteric afferents which are mechanically sensitive influencing motility, satiety, gastric emptying, and proximal stomach relaxation. The subjective result is increased appetite and meal volume [14, 15]. Stomach distention, cholecystokinin, and leptin (which bind to receptors on the vagus and nodose ganglia) produce satiety and reduced nutritional intake [16–18]. Also found on intrinsic enteric neurons are mu and kappa opioid receptors. Activation of mu receptors inhibits peristalsis, stimulates circular muscle segmentation, reduces intraluminal secretion, and increases fluid absorption from the bowel lumen [19, 20]. The use of mu agonists for visceral pain will therefore have an adverse effect on gastrointestinal motility and may increase patient symptom burden. Vagus muscle mechanicoreceptors are low threshold which elicit maximum firing rates within physiologic distention pressures and differ from extrinsic spinal afferents [21]. The mechanicoreceptors consist of “in-series-tension” receptors between longitudinal and circular muscle and intraganglionic laminar endings which are intimately arrayed around enteric ganglia. Both in series-tension receptors and laminar endings contain nonencapsulated low-threshold sensors with primitive endings responsive to nonnoxious physiologic stimuli [12, 21, 22].

### 2.2. Spinal Afferents

 Extrinsic spinal visceral afferents located in the serosa and mesentery ascend to the thoracolumbar dorsal root ganglia with parasympathetic and sympathetic fibers [21]. A large battery of pain mediators (protons, prostaglandins, interleukins anti-inflammatory cytokines, and bradykinin) are released as a result of visceral inflammation, hollow organ distention or distortion, and ischemia. These mediators bind to various ion channels or receptors found on spinal afferents. Certain channels such as the transient receptor potential ion channels (TRP), acids sensing ion channels (ASIC), and sodium channels (Na) are activated resulting in an action potential. Hence, spinal visceral afferents have promiscuous chemosensitivity [13, 21, 23]. Visceral spinal afferents have a distinctive large cell body relative to somatic afferents visualized within the dorsal root ganglia. Visceral afferents makeup 7% of sensory cell bodies within the dorsal root ganglion [13, 23]. A significant proportion of visceral spinal afferents are high-threshold or silent nociceptors which respond selectively to noxious stimuli. Spinal visceral afferent action potentials propagate centrifugal and centripetal along collaterals resulting in the release of neurotransmitters, mainly calcitonin gene-related protein (CGRP) and substance P (SP) from varicose nerve terminals which in turn modulate blood flow and enteric motor reflexes; the end result is a neurogenic inflammatory response [13, 23]. Visceral spinal afferents converge on somatic afferents within the dorsal root ganglia and dorsal horn which is thought to be the mechanism for somatic referred pain [24, 25]. Convergence also occurs among visceral afferents within the same dorsal horn segment resulting in a viscerovisceral convergence and hyperalgesia [25]. In animal models, visceral pain causes a visceromotor response observed in anterior abdominal wall musculature as a result of convergence which is used as an objective measure of nociception. This can be elicited by experimental colitis, acetic acid peritonitis, or colorectal distention. Visceral convergence leads to cross-organ sensitization in which disease in one visceral organ increases the pain arising from another visceral organ innervated by the same spinal segment [22, 24, 25]. As an example, in mice and rats, experimental colitis increases bladder sensitivity and contractions [26]. Clinical individuals with irritable bowel syndrome have a greater incidence of urinary bladder sensitivity compared with the normal population [27]. Another mechanism proposed for viscerovisceral sensitivity is dichotomized sensory fibers in which one afferent neuron innervates two visceral organs [28, 29]. Another proposed mechanism is generation of retrograde action potentials via viscerovisceral convergence leading to upregulation of neurotransmitters and ion channels in the second visceral organ [30, 31]. Finally, visceral afferents travel with parasympathetic and sympathetic fibers which activate afferents within autonomic ganglia by way of collaterals resulting in an autonomic and motor response in the second visceral organ [32]. Spinal visceral afferents have large overlapping receptive fields relative to somatic afferents and sparse innervation relative to somatic tissues. Because of the anatomical differences and convergence, visceral pain is poorly localized, diffuse, and frequently identified by its somatic referral pattern. Viscerovisceral hypersensitivity can lead to diagnostic dilemmas as to where the pathology is occurring or misdiagnosis of the origin of visceral pain [12]. Somatic convergence can lead to inhibitory or excitatory responses. A counterirritation can produce an inhibitory response at either somatic or visceral site. Inhibitory responses from convergence can last as long as one second and may account for the colicky nature of visceral pain [33]. Patients often use counterirritation at referred somatic sites that reduce visceral pain [33].

 Second-order visceral afferents ascend the thoracolumbar spinal cord through the dorsal column to gracile and cuneate nuclei. As a result, an anatomical cervical midline myelotomy interrupts dorsal column secondary afferent neurotransmission and reduces visceral pain and nocifensive responses in animal with pancreatic and duodenal pain [34–36]. Third-order afferents ascend to the ventral posterolateral and central lateral nucleus of the thalamus [37, 38]. The central lateral nucleus is the major site for medial afferents traffic important to emotive pain responses. Visceral afferents are highly represented in the medial pain matrix (insular cortex, prefrontal cortex, and anterior cingulate gyrus) which is responsible for the large emotional response to visceral pain. Visceral afferents are sparsely represented in S1 cortex; hence, visceral pain is poorly localizable by patients. The anterior cingulate gyrus is critical to downward modulating pain through the periaqueductal gray and rostral ventromedial medulla (RVM) [39]. The RVM has been demonstrated to exert descending facilitatory influences in visceral pain process and is a critical compound to the maintenance of pain from visceral inflammation [40, 41].

## 3. Important Channels, Receptors, and Mediators of Visceral Pain

 Several channels are important to visceral pain: transient receptor potential vanilloid-1 (TRPV-1), ASIC3 channels and sodium channels (Na) particularly those that are tetrodotoxin resistant (NA 1.8 and 1.9), and calcium channels. Certain receptors downmodulate pain: the gamma aminobutyric acid-B (GABA-B) channels, kappa and mu opioid receptors, and somatostatin receptors. These channels and receptors are potential targets for novel analgesics to treat visceral pain.

### 3.1. Transient Receptor Potential Ion Channels

The TRP family of ion channels is TRPV1, TRPV2, TRPV3, TRPV4, TRPM 8, and TRPA1. These channels are, in general, thermoreceptors found on poorly myelinated and nonmyelinated afferents arising from the dorsal root ganglia, nodose ganglia, and the CNS [42–46]. TRP channels also respond to nonnoxious heat (TRPV3, TRPV4, and TRPM8), capsaicin (TRPV1), methanol (TRPM8) camphor (TRPV3), mustard (TRPA1), protons (TRPV1) and the endogenous cannabinoid, and anandamide (TRPV1) [46]. These channels are calcium permeable, nonselective channels with 4 identical subunits and a central pore. Each subunit consists of a 6 transmembrane (TM) segments with an amphipathic region between TM5 and TM6 that allows cations through. The cytoplasmic N and C terminal tail of the subunits can be modulated by various kinases through phosphorylation. Phosphorylation influences channel endocytosis and expression. Chemical activators may be direct channel activators or may allosterically modify channels to reduce conduction thresholds [46, 47]. The TRP family is subject to multiple single-nucleotide polymorphisms which influence channel activation. These channels are posttranslationally modified by multiple kinases: protein kinase A, protein kinase C, calcium calmodulin kinase II, and the tyrosine kinase src which prevents channel internalization and desensitization, amplifying channel conductance. Multiple pain mediators (certain inflammatory cytokines (ILB), bradykinin, proteases, and ATP) stimulate kinase activation and channel phosphorylation [46, 48, 49].

TRPV1 channels are highly expressed on visceral spinal afferents and colocalize with nerve growth factor receptor (trk-A). They are selectively expressed on peptidergic neurons (neurons which express CGRP and SP) [42, 45]. TRPV1-activated afferents release inflammatory peptides (CGRP and SP to produce neurogenic inflammation, glutamate. TRPV1 plays a significant role in gastrointestinal inflammation, hypersensitization, but also function [50–52]. TRPV1 is expressed in bladder afferents and plays a role in micturition as well as pathologic states of interstitial cystitis [53]. Inflammatory states such as Crohn's colitis upregulate TPRV1 through kinases. Nerve growth factor and prostaglandins upregulate TRPV1 through the same mechanism [54–57]. TRPV1 has been demonstrated to be upregulated in upper gastrointestinal disorders such as gastroesophageal reflux disease and pancreatitis [58, 59]. TRPV1 remains upregulated not only in those with inflammatory bowel disease in remission but also with irritable bowel type symptoms [60]. TRPV1 is also upregulated in the CNS; inhibitors in development may need to cross the blood-brain barrier to be fully effective [45].

TRPA1 is highly expressed on primary visceral afferents and is important in visceral hypersensitivity [61]. TRPA1 is upregulated in experimental colitis and deletion of TRPA1 expression in a mouse gene “knocke-out” model reduced colitis-induced mechanical hypersensitivity [62].

### 3.2. Acid Sensing Ion Channels

ASIC family of ion channels is directly gated by protons as “chemoelectrical transducers.” Tissue acidosis with ischemia, fractures, tumor, and incisions activates ASICs at a pH below 7.4, particularly below 7.0 [63, 64]. ASIC undergoes sustained depolarization with tissue acidosis resulting in nonadapting pain [63–65]. The channel structure consists of 6 subunits derived from 4 different genes. Each subunit is identified by the intracellular C and extracellular N terminus amino acid composition (ASIC 1a, 1b, 2a, 2b, 3, and 4) [66]. Each subtype has 2 hydrophobic transmembrane domains which flank a large extracellular loop. Although ASIC channels mainly gate protons, they are also permeable to sodium. ASIC subtypes form channel dimers which influence channel expression. Homomeric ASIC 1a dimers conduct calcium [66]. Subtypes 1, 2, and 3 are expressed on intrinsic enteric neurons, and subtypes 3 are particularly expressed on spinal extrinsic afferents [67–69]. ASIC3 is up-regulated by inflammatory cytokines, metal ions, and certain neuropeptides (FMRT amide) [70]. Such neuropeptides are increased in the spinal cord and dorsal root ganglia with visceral inflammation. The endogenous kappa opioid receptor agonist, dynorphin, enhances ASIC activity and prevents desensitization [64]. Nonsteroidal anti-inflammatory drugs (NSAIDs) such as diclofenac inhibit ASIC3. Toxins from the spider, *Psalmopoeus cambridgei*, and the sea anemone, *Anthopleura elegantissima*, contain ASIC inhibitors. The potassium sparing diuretic, amiloride, also inhibits multiple ASIC subtypes [71–74].

ASIC channels are more important to visceral mechanicoreceptors sensory function than cutaneous afferent function [69, 75]. ASIC3 is also an important mediator of cardiac pain from coronary ischemia, cough, and bronchospasm from airway acidification [76, 77].

### 3.3. Voltage-Gated Sodium Channels

Sodium-gated channels can be divided into those which are tetrodotoxin sensitive (Na 1.1–1.7) and resistant (Na 1.8 and 1.9) [78]. Tetrodotoxin-resistant channels and Na 1.7 are mainly expressed on nociceptor sensory afferents [79]. These membrane glycoproteins have large alpha subunits and smaller beta subunits which form 9 recognizable subsets [80]. These channels have 4 domains made up of individual 6 transmembrane segments (S1-6). The sodium pore is between the fifth and sixth segments. Na 1.8 is the most important sodium channel visceral pain and hypersensitivity [80–82]. In experimental colitis, Na 1.8 expression is increased threefold in dorsal root ganglia within that T 9-13 segment of the mouse. There appears to be a selective expression of this channel and not other sodium channels [83, 84]. Na 1.8 channels are also important to bladder hypersensitivity caused by chemical irritants in experimental animals. Na 1.8 is not important to pain of pancreatitis [85, 86]. In the gene “knock-out” (Na 1.8 null) mouse model, experimental jejunitis failed to produce the expected hypersensitivity as measured by visceromotor responses [81, 82]. Both nerve growth factor and prostaglandins (PGE2) increased expression of tetrodotoxin-resistant sodium channels [87–89]. As a result, NSAIDs indirectly block sodium channel neurotransmission by preventing PGE2-induced upregulation of sodium channels [89]. Sodium channels can be up-regulated by phosphorylation of their intracellular C terminus [90, 91]. There are no selective Na 1.8 inhibitors which could potentially target visceral pain. There are a group of peptides, (conotoxins) that are a source of sodium channel blockers being developed, one of which has been approved for spinal analgesia [92].

### 3.4. Voltage-Gated Calcium Channels

Voltage-gated calcium channels have an alpha-1 subunit which forms the channel pore and alpha-2 delta subunit which facilitates alpha-1 traffic to membrane surfaces [93]. There are 10 different alpha-1 subunits and 4 alpha-2 delta subtypes. Alpha-2 delta subtypes 1 and 2 bind pregabalin and gabapentin. Subtype 1 is found within the medial pain matrix (anterior cingulate gyrus, insular cortex, and amygdala) [94, 95]. Alpha-2 delta subunit 1 is also expressed within the dorsal root ganglia, spinal cord, and smooth muscle of the small bowel associated with N-type calcium channels [94]. Subtype 2 is found in the periaqueductal gray, spinal cord, and diffusely throughout the CNS but not in colon or small bowel smooth muscle [95, 96]. Pregabalin and gabapentin bind to alpha-2 delta subunits in the cytoplasm and prevent expression of calcium channels on membranes [97, 98]. By binding and preventing expression, calcium conduction is blocked, and SP, CGRP, and glutamate cannot be released from primary afferent neurons [93]. Prevention of neurotransmitter release by pregabalin and gabapentin occurs only in pathologic states when calcium channels are being up-regulated and activated [99, 100]. Pregabalin and gabapentin are predominantly central acting analgesic [101–103]. Gabapentinoids have been shown to reduce visceral hypersensitivity in experimental animals as well as symptoms of irritable bowel syndrome in humans [104–109]. Low-dose gabapentin and morphine reduced writhing in rats given intraperitoneal acetic acid at doses which were found to be ineffective as single agents [110]. Not only do gabapentinoids reduce nociceptive neurotransmission centrally but also improve bowel compliance to distention perhaps through blocking alpha-2 delta subunits in smooth muscle [105, 111].

Other calcium channels may also be involved in visceral hypersensitivity. Activation of T-type calcium channels subtype Ca (v) 3.2 on primary spinal visceral afferents has been associated with irritable bowel-like symptoms in an animal model. Symptom behaviors resolved when T-type calcium channels were blocked [112]. In experimental bowel ischemia, mesenteric afferent neurotransmission was blocked by the L-type calcium channel blocker nifedipine [113].

### 3.5. Opioid Receptors

Kappa and mu opioid receptors are found on visceral afferents [114–116]. Distribution along the gastrointestinal tract indicates that endogenous opiate peptides have a modulating function for both gastrointestinal motility and secretory function. Constipating effects and dysmotility with morphine is predominantly due to actions on intrinsic enteric neurons [117]. Kappa receptors have little adverse effect on gastrointestinal motility, but doses are limited by central toxicity such as dysphoria [118–120]. The central toxicity of mu receptor agonists includes confusion, myoclonus, nausea, and vomiting which limits titration and adds symptom burden to patients with visceral pain [118]. In addition, certain mu opiates such as the enkephalin derivative DAMGO stimulate mesenteric afferents action potentials which are blocked by the opioid receptor antagonist alvimopan [121]. Mesenteric afferents respond with increasing firing rate when exposed to mu and delta receptor agonists which does not occur with selective kappa receptor agonists [122, 123]. Morphine has been shown to increase the visceral hypersensitivity and viscerosomatic referral in animal models of colitis and irritable bowel syndrome (caused by butyrate) [124].

There are 3 subtypes of kappa receptors which inhibit afferent firing and visceromotor responses to noxious colorectal distention in animal models [125–129]. In these animal models, antinociception occurs without change in colon muscle compliance [125]. Kappa opioids in animal models reduced inflammation (colitis) nociceptive responses [130–132]. Kappa receptor gene “knocked-out” mice display increasing writhe to acetic acid peritonitis compared with wild-type mice [133]. The kappa receptor agonist, CR 66, in humans selectively reduced visceral pain by esophageal distention pain tolerance but reduced somatic pain thresholds as measured by cutaneous pain [134]. Kappa receptors are important modulators of visceral pain, and it is the peripheral kappa receptor not the central receptor which is important in contradistinction to mu receptor agonists.

Kappa receptors are also found on the vagus and nodose ganglia. The kappa agonist, asimadoline, has been reported to reduce satiety and enhance postprandial gastric volumes [135, 136]. Asimadoline has no influence on motility. Asimadoline reduced pain in a group of patients with irritable bowel syndrome [136]. However, “on-demand” asimadoline was not effective in reducing irritable bowel symptoms and worsened symptoms in diarrhea prone patients [137]. A second peripheral-restricted kappa receptor agonists, fedotozine, reduced nociceptive reflexes triggered by gut distention and reduced pain related to irritable bowel syndrome and nonulcer dyspepsia [138]. Fedotozine reversed ileus caused by acetic acid peritonitis in rats and prevented c-fos expression in the spinal cord, a measure of nociceptive traffic through the dorsal horn. C-fos was also reduced in numerous upstream brain structures demonstrating the importance of blocking nociceptive traffic to prevent neuroplasticity [139]. Fedotozine also did not influence gastrointestinal transient times. Fedotozine was ineffective in reducing symptoms and gastroparesis in diabetic patients [140]. The kappa receptor agonist ADL 10-0101 reduced pain in patients with pancreatitis without influencing heart rate, respiratory rate, oxygen saturation, or causing nausea. Analgesia appeared to be mediated by receptors outside of the CNS [141]. Other peripherally restricted kappa agonists are being developed [142].

DAMGO and morphine are more potent in reducing acetic acid peritonitis in experimental animals when given intracerebroventricular than intraperitoneal. Morphine antinociception was only partially reversed by peripherally restricted mu receptor antagonists [143]. Methylnaltrexone, a peripherally restricted mu receptor antagonist, reverses dysmotility morphine and other mu opioid agonists without reverse seen analgesia [144, 145]. Mu receptor agonists such as fentanyl are reported to reduce visceromotor responses to colorectal distention in mice [146]. Neither morphine, fentanyl, or delta agonists reduced the firing ratio of mesenteric afferents, whereas kappa receptor agonists (U-50,488 and fedotozine) did reduce firing rate suggesting that the opioid-induced sensitization occurs centrally and that mu receptors are either absent on extrinsic spinal afferents or do not influence action potentials [147]. These findings again indicate that central and not peripheral mu receptors are critical to modulating visceral pain; kappa agonists mediate pain relief by binding to peripheral receptors.

There is evidence that fentanyl significantly reduces visceromotor responses to a greater extent when kappa receptors are nonfunctional (kappa receptor gene “knocked-out” mice) [148]. The combination of fentanyl and the kappa receptor agonist spiradoline produced dose-dependent supra-additive antinociception in an animal model involving colorectal distention. However, there appears to be a complex interplay between kappa and mu receptors. Fentanyl nociception was not blocked, but the kappa agonist antinociception was blocked by the mu receptor antagonist (b-FNA) perhaps suggesting an interaction through dimers [149]. There is little data about the benefits of combining kappa and mu receptor agonists in the clinical management of visceral pain. A recent study of human volunteers found that oxycodone which is thought to be a kappa and mu receptor agonist significantly blocked visceral pain better than morphine which has little kappa receptor activity [150]. By speculation, a combination of a mu receptor agonist and a peripherally restricted kappa receptor agonist might selectively improve visceral pain relative to a peripherally restricted kappa agonist or mu agonist alone.

### 3.6. Gamma Aminobutyric Acid Receptors

Gamma aminobutyric acid receptor-B (GABA-B) is involved in modulating mechanicosensory traffic through the vagus and brainstem pathways. GABA-B receptors block mechanico-sensory input but do not modulate chemosensitivity. GABA-B agonists directly influence sensory input via binding enteric receptors or by way of activating inhibitory interneurons within the CNS [151, 152]. Baclofen, a GABA-B receptor agonist, has been used in animal models to test visceral nociception. GABA-B receptors couple with N and P/Q calcium channels and potassium channels which may be the mechanism of antinociception. Activation of receptors downregulates calcium channels and activates inward rectify potassium channels which improves repolarization of neurons [153–155]. Baclofen in male long Evans rats reduced afferent firing from a colorectal distention. This occurs in a dose-dependent manner unrelated to smooth muscle relaxation or improved colon compliance [156, 157]. Visceromotor responses from colorectal distention are reduced by baclofen [157–159]. Baclofen also blocks the expression of c-fos in the dorsal horn to experimental colitis and capsaicin-induced bladder irritation [160, 161]. Baclofen reduced presynaptic release of SP from visceral afferents [162]. Volatile anesthetics such as halothane, isoflurane, sevoflurane sevoflurane, and propofol block visceral nociception via augmentation of GABA-B [163, 164]. Baclofen in most clinical studies was intrathecal, and the pain phenotype was largely neuropathic [165–169]. Because baclofen crosses the blood-brain barrier, benefits may be mediated centrally. There is some evidence for synergy between morphine and baclofen [166], but very little clinical data are available about the use of baclofen for visceral pain.

### 3.7. N-Methyl-D-Aspartate Receptors and ATP-Gated Ion Channels

N-Methyl-D-aspartate (NMDA) receptors are calcium channels activated by glutamate and are slowly desensitized once activated. These high-calcium permeable channels generate synaptic neuroplasticity, wide dynamic range neuronal responses, and gene expression within the CNS [170, 171]. NMDA receptor activation has been associated with visceral hyperalgesia [170]. Due to slow inactivation, NMDA receptors produce long-term potentiation (LTP) which generates chronic pain [172]. NMDA receptors are found on enteric extrinsic afferents from colon and bladder; these afferents release CGRP and SP once the receptor is activated [173]. NMDA receptors are also found on postsynaptic, second-order visceral afferents, within the brainstem and cortex [174–178]. Receptor activation by colon distention does not require inflammation to be present which is distinctly different from somatic NMDA receptors [179–181]. Intravenous but not intrathecal memantine (an NMDA receptor blocker) reduced visceromotor responses to colorectal distention suggesting that peripheral NMDA receptors are important to antinociception [173]. The NMDA receptor blocker, MK-801, prevented dorsal horn expression of c-fos to noxious colorectal distention, and another NMDA receptor blocker, APV, attenuated visceromotor responses to graded colorectal distention in a dose-dependent manner [173]. NMDA receptors within the RVM are important in modulating pain [182].

There are 2 purinergic ion channels, P2X and P2Y. P2X is an ATP-gated channel, and P2Y is a G-protein couple receptor. ATP released by cell damage, from the sympathetic nervous system or extrinsic sensory neurons, binds to P2X. A subset of P2X channels (P2X3) were upregulated in extrinsic afferents neurons by inflammatory bowel disease [183]. ATP released from the epithelial lining of the mucosa binds to several subsets of P2X (P2X3 and P2X2/3) on submucosal sensory neurons. In this way, P2X modulates peristalsis [184–186]. P2X, upregulated by interleukin-1b, was found to be a mechanism for postinfectious (jejunitis) hypersensitivity in animal models. Animals not expressing P2X subtype 7 on afferents did not develop postjejunitis visceral hypersensitivity [187]. 

### 3.8. Somatostatin and Bradykinin Receptors

Octreotide blocks somatostatin-2 receptors (SST-2). Mesenteric afferent firing rates caused by ramping jejunal pressures in Wistar rats were blunted by octreotide [188]. Reduced firing rates occurred without change in small bowel compliance. Octreotide also blocked bradykinin activation of mesenteric afferents and improved symptom behaviors of irritable bowel syndrome in animals [188–190]. Somatostatin influences motility and increases gastrointestinal transit time [191]. Octreotide anecdotally reduced the abdominal pain of Menetrier's disease and pain related to visceral cancer metastases [192, 193]. Octreotide as an adjuvant to morphine reduced pain in a cohort of individuals with gastrointestinal cancers [194]. Octreotide relieved severe refractory epigastric pain in individuals with weight loss and functional bowel disorders [195]. Unfortunately, octreotide did not reduce pain from pancreatitis [196].

Bradykinin activates mesenteric afferents by way of B2 receptors and by stimulating prostaglandin production [197, 198]. The herbal preparation STW-5 (Ibergast Steigerwald Gmb H, Darmstadt, Germany) dosedependently blocks bradykinin-induced afferents discharges from mesenteric sensory neurons [199–201]. Ibergast was effective in reducing symptoms from functional dyspepsia as reported in a meta-analysis [202]. Ibergast also alters smooth muscle contraction and motility [203, 204].

## 4. Protein Kinases

Protein kinase A phosphorylates NMDA receptor subunit NR-1 and the transcription factor cyclic AMP (cAMP) response element binding protein (CREB). CREB upregulates the receptor for SP (neurokinin-1 receptor) [205, 206]. Colonic distention induced protein kinase A phosphorylation of NMDA receptors in the second-order postsynaptic dorsal column neurons. This was be blocked by the NMDA receptor antagonist, CNQX [37]. In the same manner, protein kinase C is upregulated in the gracile nuclei and in postsynaptic dorsal column neurons with visceral stimulation. Protein kinase C phosphorylates a second glutamate receptor, AMPA [207]. Formalin-induced colitis increased the activation of protein kinase C through attachment to neuronal membranes in the dorsal horn [208]. The mitogenic activated protein kinase family of kinases regulates neuron responses and neuroplasticity. Within this family, the extracellular signaling-related kinases (ERK 1, 2) are important to visceral pain. Activation of ERK has been associated with visceral hyperalgesia [209]. ERKs couple to multiple receptors including NMDA and neurokinin-1 [210, 211]. Increased expression ERK 1, 2 in the L6-S1 mouse dorsal horn was associated with a leftward shift in visceromotor responses to colorectal distention. Blocking this kinase attenuated visceromotor responses [212].

## 5. Pharmacologic Management of Visceral Pain

Clinical trials of specific analgesics for visceral pain are sparsely published. Visceral pain is included with somatic and nociceptive pain in most clinical trials; as a result, it is difficult to determine the appropriate drug choices for visceral pain as a phenotype. The fact that there are some differences between somatic and visceral pain neurotransmission, neurotransmitters, channels, and receptors suggests that there may in fact be real differences in responses to analgesics. The use of potent opioids for inflammatory bowel disease has been associated with higher morbidity and mortality which is not reported for various types of somatic pain [213]. This may reflect the fact that patients on opioids are sicker than those who are not but this may also be related to the opioid. In a similar fashion, certain NSAIDs have been associated with poorer outcomes in inflammatory bowel disease but not for somatic pain [214, 215]. On the other hand, octreotide improves colic and symptoms from bowel obstruction better than anticholinergics; potent opioids actually may worsen colic [216, 217].

It is unlikely that a single analgesic or targeted agent will significantly reduce most visceral pains since multiple neurotransmitters, channels, and receptors are responsible for visceral pain. Analgesics combinations are anticipated to be better than single analgesics [218, 219]. Analgesics which are ineffective as single agents or ineffective at a particular dose may have supra-additive analgesia for visceral pain when combined with a second analgesic. Hence, single-drug trials may not predict the merit of an analgesic supra-additive analgesic combinations cannot be predicted based on single-drug activity. Analgesic dose response relationships for visceral pain may be distinctly different from somatic pain [220]. Opioids for somatic pain may in fact not work well for visceral pain and visa versa. Kappa agonists may be an example [150, 221].

Gastrointestinal motility has been assumed to be a surrogate for gastrointestinal symptoms [222]. For instance, it has been assumed that early satiety is related to reduced gastric emptying or poor proximal gastric compliance. However, kappa receptors improve satiety without changing gastrointestinal motility. Morphine and its metabolite, morphine-6-glucuronide, reduce visceral pain but increase circular muscle contraction, whereas somatostatin reduces visceral pain and reduces smooth muscle contraction [223–226]. TRPV1, Na 1.8, ASIC3 targeted analgesics, and kappa receptor antagonists may have less adverse effects on gastrointestinal motility than somatostatin or mu agonists though future trials will need to confirm this speculation.

Another point is that selective visceral blocks and procedures (celiac, hypogastric, and splanchnic blocks, spinal cord stimulators, radiofrequency ablation, and midline myelotomy) needed to complement medical management [35, 227–229]. Multimodality approach for pain management requires a multidisciplinary group of experts. There is a tendency to overly rely on analgesics and refer individuals to other disciplines late when pain is poorly controlled; the cancer is widespread and patient tolerance to procedures is poor [230].

There is little doubt that central sensitization plays a role in maintaining visceral pain [79, 182, 231–233]. As a result, uncontrolled acute visceral pain will likely lead to central neuroplasticity and chronic pain despite the resolution of underlying cause for pain. Certain analgesics will need to penetrate the CNS to fully and effectively relieve pain [182].

### 5.1. NSAIDs and Paracetamol

Paracetamol is a weak cyclooxygenase-2 inhibitor and a selective cyclooxygenase 3 inhibitor. It also increases brainstem serotonin neurotransmission, redirects beta-endorphin, and inhibits 5-HT 3 receptors which are pronociceptive [234–237]. Paracetamol is commonly used for pain, but little is known about visceral pain responses since most studies have not focused on visceral pain. In an acetic acid peritonitis model, combinations of ketoprofen plus paracetamol were synergistic at a dose ratio of 3:1 (paracetamol to ketoprofen) [238]. Several other studies involving animal models of visceral pain have confirmed benefits of NSAIDs plus paracetamol combinations [239, 240]. In a qualitative systematic review of paracetamol plus NSAIDs combinations, combinations were superior to paracetamol alone in 85% and NSAIDs alone in 64% of studies. Ibuprofen was the most commonly used in the 21 studies reviewed. Drug combinations reduced pain by 35% and analgesic supplemented by 38%. This combination was superior in gynecology surgical studies (83%) indirectly confirming benefits for visceral pain [241].

NSAIDs are effective in reducing cancer pain in the dose-dependent fashion [242]. However, a major systematic review was not selectively focused on visceral pain [243]. Multiple studies have found that NSAIDs are effective for biliary colic and are as effective as opioids with fewer side effects [244–247]. One of the mechanisms by which NSAIDs work in renal or biliary colic may involve acetylcholine blockade. Diclofenac blocks acetylcholine-induced smooth muscle contraction [248]. NSAIDs were superior to anticholinergics in relieving biliary colic with a number 3 needed to treat and prevented progression to cholecystitis better than anticholinergic with a number 3 needed to treat [249, 250]. In the same vein, NSAIDs are superior to anticholinergics in relieving renal colic [251]. In a meta-analysis, NSAIDs were superior to opioids in reducing renal colic [252]. Both NSAIDs and opioids provide effective analgesia in acute renal colic, but opioids are associated with a higher incidence of adverse events, particularly vomiting [252].

Nine percent of individuals with inflammatory bowel disease treated with NSAIDs deteriorate clinically and improve once the NSAID is discontinued. Loss of prostaglandin by the NSAID leads to microvascular dysfunction and sustained inflammation [215, 253, 254].

### 5.2. Opioids

Opioids reduce pain; however, poor coping skills, depression, and catastrophization correlate better with dose than the degree pathology [255, 256]. Opioids add a symptom burden associated with dysmotility [19, 257, 258]. Proactive use of laxatives and bowel softeners are needed. In addition, morphine can produce visceral hyperalgesia [124, 259] which is suppressed by gabapentinoids [260]. This may explain the advantages of this combination when treating visceral pain [219]. Features of hyperalgesia are increased pain intensity extent of pain area, or radiation with reduced responsiveness to opioid analgesia [261]. Opioid-induced hyperalgesia mimics pain associated with progression of the underlying pathological condition. Physicians unaware of this phenomenon increase the opioid dose only to worsen visceral pain. The addition of adjuvant analgesics or opioid rotation is preferred in managing opioid-induced hyperalgesia [262, 263]. Opioid functional selectivity occurs in multiple downstream signaling pathways (beta arrestin 1 and 2, kinases, and G proteins) caused by unique opioid-receptor conformation changes [264]. Functional selectivity is also determined by expression of certain proteins within the vicinity of the receptor. Opioid rotation will alter downstream signaling and reset the analgesic response. Paradoxically, opioid dose reduction may improve pain control [265].

Sustained release oxycodone significantly reduces chronic cancer-related visceral pain. In a multicenter prospective observational study involving 350 individuals with visceral pain, oxycodone reduced pain severity from a mean of 7 to a mean of 2.4 on a numerical rating scale (0 no pain, 10 severe pain) over a two-week period of time. Less than 5% of individuals continued to have severe pain at the end of 2 weeks [266]. A small randomized trial found that morphine was as effective as oxycodone in relieving pain related to pancreatic cancer [267]. Intranasal fentanyl was effective in managing acute visceral pain in the emergency department [268]. At the present time, there is little published data to give direction to the choice of opioids for visceral pain.

Combinations of analgesics which include opioids have been reported in the management of visceral pain. In an animal model, blocking TRPV1 enhanced opioid antinociception [269]. In a mouse model, the triple combination of fentanyl, trazodone, and paracetamol reduced writhing to acetic acid at doses lower than those seen with single drugs [270]. In the rat colorectal distention model, ketorolac alone was ineffective, and morphine modestly effective but the combination of ketorolac and morphine produced synergistic antinociception at dose ratios of 10:1 and 20:1 [271]. Combinations of tramadol plus morphine and tramadol plus fentanyl produced synergistic antinociception in the acetic acid writhe model but not with somatic pain (hot plate test) [272].

### 5.3. Adjuvant Analgesics

There is preclinical and some clinical evidence that adjuvant analgesics should be used early for visceral pain. The combination of a gabapentinoid plus opioid is a reasonable choice [109, 219]. Octreotide reduces visceral hyperalgesia which is a logical choice to use with morphine in malignant-related bowel obstruction [189]. STW-5 blocks bradykinin receptors and pain from jejunal distention and could potentially improve opioid analgesia [200]. Baclofen reduces visceral nociception in multiple models which makes it attractive to use with opioids and nonopioid analgesics (NSAIDS or paracetamol) [156]. NMDA receptor antagonists reduced visceral pain in a different manner advanced somatic pain and may improve the opioid analgesia for visceral pain differently than from somatic or neuropathic pain [173]. Combinations of NSAIDs, paracetamol, and opioids are likely to be at least additive if not synergistic [241]. Dexmedetomidine, an alpha-2 antagonist, inhibited visceromotor responses to colorectal distention [273]. In the same manner, tricyclic antidepressants and serotonin norepinephrine reuptake inhibitors could improve visceral pain by increasing norepinephrine neurotransmission. In addition, tricyclic antidepressants inhibit sodium channels, NMDA receptors, P2X channels, and prostaglandins [274]. Combinations of antidepressants and opioids may improve visceral pain in a similar fashion as the combination does for neuropathic pain. Overall, the clinical evidence for drug combinations is weak and requires prospective clinical trials.

## 6. Summary

Visceral pain is mediated by unique peripheral and central pathways. Several ion channels TRPV1, Na 1.8 and ASIC3, and the kappa opioid receptor appear are particularly important to modulating pain. There are qualitative and quantitative differences in pain processing from somatic pain such that visceral pain should be considered a distinct phenotype. Drug development and treatment paradigms have to take this into consideration in cohort and randomized trials. This has been done successfully for the irritable bowel syndrome. Targeted agents are being developed to ion channels; it is unlikely that these targeted agents will produce less dysmotility than mu opioid receptor agonists. Ion channel blockers are likely to have only a modest benefit due to the multiplicity of ion channels involved in visceral pain and will have to be combined with other analgesics. There is evidence that targeted agents for TRPV1 channels improve opioid responses. Adjuvant analgesics such as the gabapentinoids are effective as single agents and should be considered early in the course of opioid therapy to prevent opioid-induced hyperalgesia and minimize opioid side effects by being “opioid sparing.” Peripherally restricted kappa agonists are in development and should be combined with other analgesics and adjuvants in hope of improving pain control. Interventional procedures should be considered early to avoid chronic visceral pain poorly responsive to analgesics. Treatment algorithms have been developed for neuropathic pain based on multiple randomized trials. The same approach should be taken in the management of visceral pain.

## Figures and Tables

**Table 1:** Distinctive features of visceral spinal afferents relative to somatic.

(1)	Dual extrinsic afferent system
(2)	Convergence of afferents on somatic and other visceral afferents within the spinal cord
(3)	Widely overlapping receptive fields
(4)	Dichotomization of fibers where a single neuron innervates two viscera
(5)	Collateral activation of autonomic and enteric nervous system
(6)	Larger cell bodies within dorsal root ganglia
(7)	Wide overlapping receptor fields
(8)	Lack of specialized nerve terminals
(9)	First-order afferents arborize over several spinal segments
(10)	Greater expression of transient receptor potential (TRPV_1_), sodium (Na 1.8), acid (ASIC3) ion channels, and calcitonin gene-related protein (CGRP)
(11)	Limited number of stimulus responses (distension, ischemia, and inflammation)
(12)	More discrete location of first-order terminals within the spinal cord (superficial dorsal horn, lamina V, and central)
(13)	Afferents ascend with parasympathetic and sympathetic neuronal projections
(14)	Viscerovisceral hyperalgesia and hypersensitivity
(15)	Visceromotor responses and referred pain to somatic sites innervated by the samespinal cord level
(16)	Second-order afferents ascend in the dorsal column
(17)	Nonsomatotopically arranged input in dorsal column and central lateral thalamus unlike the lateral spinothalamics
(18)	Poor representation in S1 cortex
(19)	Greater emotional and autonomic responses to pain than somatic pain
